# *In Situ* Elevated Temperature Testing of Fly Ash Based Geopolymer Composites

**DOI:** 10.3390/ma9060445

**Published:** 2016-06-03

**Authors:** Les Vickers, Zhu Pan, Zhong Tao, Arie van Riessen

**Affiliations:** 1 Geopolymer Research Group, John de Laeter Centre, Curtin University, GPO Box U1987, Perth WA 6845, Australia; vickers4@bigpond.net.au; 2 Institute for Infrastructure Engineering, Western Sydney University, Penrith NSW 2751, Australia; z.pan@westernsydney.edu.au (Z.P.); z.tao@westernsydney.edu.au (Z.T.)

**Keywords:** fly ash geopolymers, alumina aggregate, *in situ* thermal testing, fire testing

## Abstract

*In situ* elevated temperature investigations using fly ash based geopolymers filled with alumina aggregate were undertaken. Compressive strength and short term creep tests were carried out to determine the onset temperature of viscous flow. Fire testing using the standard cellulose curve was performed. Applying a load to the specimen as the temperature increased reduced the temperature at which viscous flow occurred (compared to test methods with no applied stress). Compressive strength increased at the elevated temperature and is attributed to viscous flow and sintering forming a more compact microstructure. The addition of alumina aggregate and reduction of water content reduced the thermal conductivity. This led to the earlier onset and shorter dehydration plateau duration times. However, crack formation was reduced and is attributed to smaller thermal gradients across the fire test specimen.

## 1. Introduction

Geopolymers are inorganic materials produced by alkali activation of suitable amorphous precursors such as metakaolin, fly ash and volcanic ash. They are based on aluminate and silicate tetrahedra, which can combine to form a range of compositions [1,2,3]. Geopolymers are generally recognised as being superior to ordinary Portland cement (OPC) based systems with respect to thermal resistance. Changes occurring in OPC systems are considered to be irreversible after heating beyond 500 °C. This is attributed to the dehydration of OPC systems with formation of calcium oxide. On rehydration of the heated material, calcium hydroxide is reformed, which is an expansive process leading to the formation of cracks and reduced strength [4,5]. In the case of OPC, this loss of water from the hydrates present results in the reduction of strength and consequent load bearing ability [6,7].

Contrastingly, the majority of the water in geopolymer systems is not chemically bound, but is present in the pore system. The ease of escape of this water during thermal exposure is dictated by the inter connectivity of the pore system [8]. Ma *et al.* [9] demonstrated the higher porosity content and increased permeability of fly ash based geopolymer pastes compared to OPC paste. The increased pore volume connectivity of geopolymers compared to OPC during high temperature exposure will markedly increase transport of water through the binder with associated reduced spalling [7,10].

The complex chemical composition of fly ashes and the resulting geopolymers results in the formation of new phases during alkali activation and on subsequent thermal exposure [11]. The effect of these new phases and their evolution during thermal exposure needs to be clearly understood [12,13,14].

To develop thermally resistant geopolymer systems based on fly ash, knowledge of the bulk chemical and phase compositions is required. Bulk chemical composition (from X-ray fluorescence spectrometry) and crystalline phase composition (from quantitative X-ray diffraction) determination is necessary to arrive at the amorphous silicon to aluminium ratio (Si:Al) [15]. From a geopolymer synthesis perspective this amorphous Si:Al value is crucial to the development of thermally resistant fly ash geopolymer systems [16,17].

In order to optimise thermal properties, control of thermal expansion and retention/development of physical properties after elevated temperature exposure is desirable. The use of thermally stable filler material to optimise thermal properties is common place in other materials technology. Additionally the use of fibres to modify crack initiation and propagation mechanisms can play an important role in the optimisation of properties during thermal exposure.

Silva *et al.* [18] used naturally occurring wollastonite fibres (aspect ratio of 10 to 20:1) to reinforce a metakaolin based geopolymer. Toughness of the system increased as fibre volume increased to 5% by volume. The wollastonite is compatible with the pH levels in geopolymer synthesis and a dense interfacial transition zone, matrix-fibre, was developed.

Dias and Thaumaturgo [19] investigated the addition of basalt fibres (45 mm × 9 μm diameter) to metakaolin based geopolymer and OPC pastes at 0.5% and 1% volume fraction. The basalt fibres were more efficient at reinforcing the geopolymer system than the OPC system. This could probably be related to the alkali initiated bond formation between fibre and matrix [20].

Polypropylene fibres are used sacrificially, melting and vaporising on exposure to elevated temperatures to form channels to facilitate water escape [21,22,23]. This results in reduced spalling tendencies of thermally exposed systems.

The ability of geopolymer systems to give controlled water release, together with inherent heat resistance, very low smoke and toxic gas emissions indicates that fly ash based geopolymers will show improved fire resistance compared to OPC and organic binders.

In the case of geopolymers and OPC, no incomplete combustion products are formed during a fire so flashover times are theoretically infinite. Organic polymers used as binders for fibre reinforcement in composites generate smoke (incomplete combustion products), which lead to flash over times of 10 to 25 min. Flashover is a phenomenon unique to compartment fires, where products of incomplete combustion collect at the ceiling and then ignite leading to total involvement of the compartment materials and signaling the end of human survivability. The time to flashover is the available escape time. The Federal Aviation Administration (FAA) has used time to flashover of materials in aircraft cabin tests as the basis for acceptance criteria for commercial aircraft cabin materials [24].

Pan *et al.* [25] compared two different Class F fly ashes in a geopolymer mortar (sand:fly ash = 3:1) using 50 mm diameter × 100 mm high cylinders. They measured strength gains and losses after exposure to 800 °C. The rate of temperature increase was 4.4 °C per minute with a two hour residence at the target temperature followed by natural cooling in the furnace. One fly ashshowed strength gains after high temperature exposure whilst the other showed losses. They suggested that further geopolymerisation at high temperatures and/or sintering could lead to gains whilst damage due to thermal incompatibility arising from non-uniform thermal gradients led to strength losses. The following was suggested as a mechanism of geopolymer thermal degradation:

1. Thermal incompatibility between matrix and aggregates
2. Pore pressure effects
3. Phase transformations

Kong and Sanjayan [26] investigated the effect of heat on geopolymer paste, mortar and concrete. Aggregates with different Coefficients of Thermal Expansion (COTE) were evaluated together with the influence of specimen size. They concluded that strength loss in geopolymer concrete at high temperature can be attributed to a mismatch in COTE between geopolymer paste and aggregate. Larger aggregate gave better strength retention. Larger samples showed lower strength retention, which was attributed to larger temperature gradients.

Pan and Sanjayan [27] described the equipment used to measure *in situ* physical properties of geopolymer paste at high temperatures. The level of preload influenced the thermal expansion behaviour in terms of absolute strain, but did not affect any deflection points with respect to temperature. A glass transition temperature of 560 °C for the paste system was determined. Above this temperature, significant viscous flow occurred as shown by the large strain increases (1200%) up to 680 °C.

Previous work [28] showed that when the silicon to aluminium, ratio was reduced below 2.0 for Collie fly ash geopolymers with the addition of 10% by volume of thermally stable fillers (α-alumina, mullite and wollastonite), improved thermal resistance was demonstrated. In this paper the work is extended with the use of high volume loadings of graded alumina together with a fibre tri-blend in the 1.82 Si:Al binder. High temperature *in situ* testing of this composite is described together with the outcomes of simulated fire testing.

## 2. Experimental Details

### 2.1. Materials

The fly ash used in this work was sourced from the Collie Power Station, Western Australia. The compositional information has been detailed previously [28]. Table 1 shows the differences in bulk and amorphous molar Si:Al. The graded α-alumina with an Al_2_O_3_ content >99 wt % was sourced from Doral Minerals, Rockingham, Western Australia. The graded sizes were selected and combined in ratios to enable processable composites to be utilised in specimen preparation. Wollastonite, NYAD MG, was sourced from NYCO Minerals, Willsboro, NY, USA. The alkaline activating solution was based on sodium silicate, PQ-D A53 (PQ Australia, Dandenong, Australia) and sodium hydroxide pellets (Rowe Scientific, Wangara, Australia). Deionised water was used to prepare sodium hydroxide solutions. Sodium based activation was selected to enable comparison with previous work. The composition of the PQ-D A53 is 44.1 wt % solids with a SiO_2_:Na_2_O modulus of 2. Adfil Ignis, monofilament polypropylene fibres with a melting point of 165 °C, were sourced from Reoco Performance Fibres, Smeaton Grange, New South Wales, Australia. They were 6 mm long and 18 μm diameter with a tensile strength of 600 MPa. The basalt fibres used in this work had a melting point of 1050 °C and were sourced from Technobasalt, Kyiv, Ukraine. They were 5 mm long and 18 μm diameter with a tensile strength of 2900 MPa. The surface of the basalt fibres was treated with 1% by weight of silane type size to improve handling characteristics.

### 2.2. Geopolymer Synthesis

The geopolymer matrix was synthesised with a targeted Si:Al ratio of 1.82, a Na:Al ratio of 1.08 and a water content of 20 wt % using a sodium hydroxide/sodium silicate solution as the activating medium. The fly ash and activating solution were mixed in a planetary mixer for five minutes prior to the addition of graded aggregate and fibres, followed by a further five minutes mixing for paste and a further seven minutes for aggregate filled composite. Table 2 shows the formulations used in this work. The geopolymer reaction mixture was poured into moulds of the required geometry, which were then vibrated to remove entrained air. The filled moulds were then sealed and cured for 24 h at 70 °C. On removal from the curing oven moulds were kept sealed for 2 days at ambient temperature (23 ± 2 °C) prior to demoulding.

### 2.3. Mechanical Testing 

**Figure 1:**
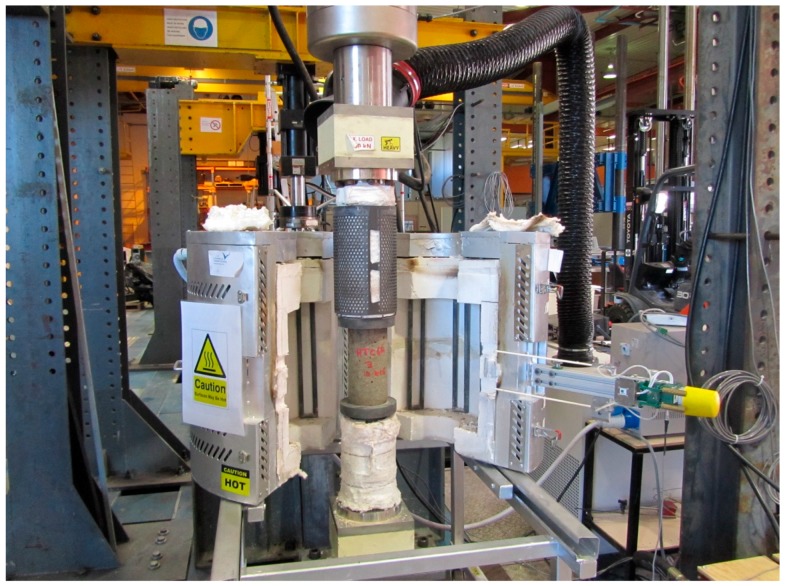
High temperature testing arrangement.

Compressive strength at high temperatures was assessed by using a thermal steady-state test method while creep at high temperatures was assessed by using the transient-state test method. All the tests were carried out using a MTS (Winchester, VA, USA) equipped with an electric split tube furnace and extensometer fitted with alumina probes (Figure 1). For hot strength tests, the specimens (without any load) were heated at a constant heating rate of 5 °C/min. Once the target temperature was reached, the furnace temperature was held for around 2 h until the specimen temperature reached a steady state. Then the specimen was loaded until failure. The procedure of the tests complied with RILEM TC 129HT [29]. For creep tests, the specimens were first loaded in compression to a stress level of 0.2σ, where σ is the reference strength at room temperature prior to heating. Then the stressed specimens were heated at a constant rate of 5 °C/min until failure occurred. During heating, the strain was measured in the transient-state by a high-temperature extensometer. The procedures proposed by RILEM [30] were followed for these types of measurements.

### 2.4. Dilatometry

A DI-24 Adamel Limohargy, Roissy-en-Brie, France (alumina push rod dilatometer) was used to determine the thermal expansion and shrinkage on 4.7 mm diameter × 15 mm long geopolymer paste rods. In the case of aggregate filled composites a 10 mm × 12 mm × 22 mm rectangular block was cut from moulded cylinders. The measurements were made over the range 25 to 1000 °C at a heating rate of 5 °C/min. A pre-load of 100 mN was applied to the sample prior to heating to allow data from shrinking samples to be collected in order to comply with ASTM E228-11 [31]. The intersection of tangents to the curve in sections of large change was used to determine the inflexion points in the data [32].

### 2.5. SEM

Scanning electron microscopy (SEM) was performed on a NEON 40EsB field emission SEM (Zeiss, Oberkochen, Germany) or MIRA3 TECSAN (Brno, Czech Republic). The platinum coated samples were then imaged in the SEM with an accelerating voltage of 5 kV using secondary electron (SE) or back scattered electron (BSE) imaging.

### 2.6. Fire Testing

A custom designed electric furnace was used for the fire testing based on Australian Standard AS1530.4. The equipment and methodology has been described previously by the authors of [33]. The time *versus* temperature relationship of this fire curve is described by Equation (1).

(1) $$T=345\log_{10}\left(8t-1\right)+20$$

where *T* = temperature (°C), and *t* = time (min).

## 3. Results and Discussion

### 3.1. Dilatometer Tests

**Figure 2:**
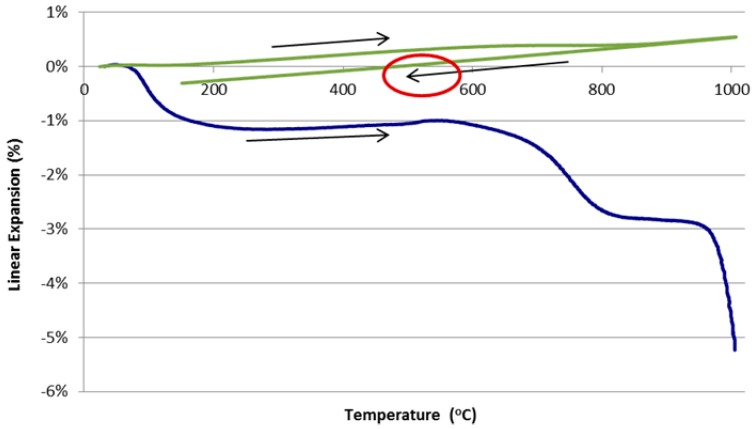
Dilatometer curves for C1.82 paste (blue) and HTC 6A composite (green). The composite curve shows both heating and cooling cycles as indicated by the direction of the arrows.

Figure 2 shows the thermal expansion curves for C1.82 (paste) and HTC 6A (composite). The large change in shrinkage at approximately 600 °C for the C1.82 specimen is caused by viscous flow and sintering and at 950 °C by further sintering and formation of crystalline phases [16,34].

The dramatic reduction in shrinkage at 1000 °C associated with the addition of 51 vol % of graded alumina to the paste is clearly evident. Shrinkage was reduced from −5% to +0.5%. The smoothing out of the composite curve and reduction in expansion range (relative to the paste) contributed to reduced crack initiation and propagation compared to the paste system. The weight (water) losses during the heating cycle reflect the total water added (Table 3). The bulk of the water was lost up to 250 °C as it resided as free water in the pore system.

### 3.2. In Situ Compressive Strength Testing

**Figure 3:**
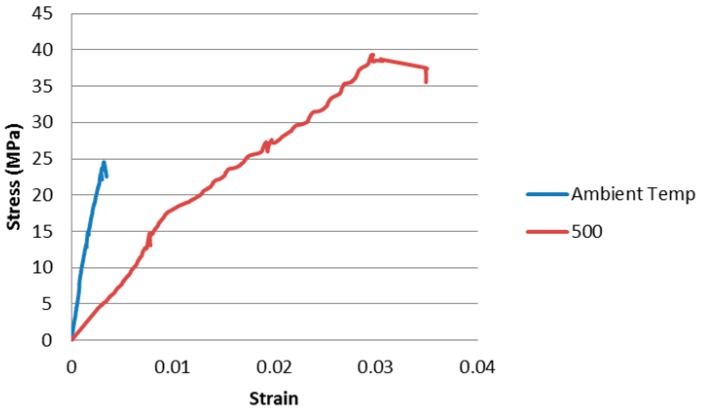
C1.82PP compression test curves undertaken at ambient and 500 °C.

**Figure 4:**
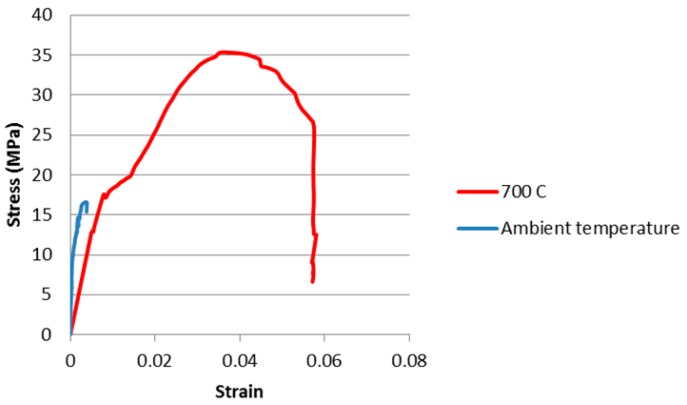
HTC 6A compression test curves, undertaken at ambient and 700 °C.

Testing temperatures of 500 and 700 °C were selected from the dilatometer curves (Figure 2). These test temperatures are 100 °C lower than the onset temperature of viscous flow obtained from the dilatometer curves. Table 3 summarises the results of the *in situ* testing while Figure 3 and Figure 4 show the stress-strain curves.

Figure 3 shows that the effect of testing C1.82PP at 500 °C compared to ambient temperature was to give a marked reduction in compressive stiffness shown by Young’s moduli of 9.8 and 2.08 GPa, respectively, and a ten-fold increase in strain at maximum load (0.003 and 0.029).

**Figure 5:**
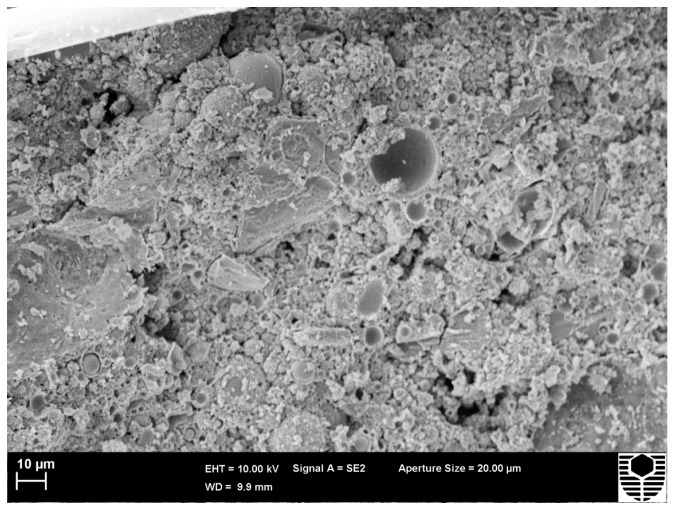
C1.82PP as cured (AC) fracture surface image.

**Figure 6:**
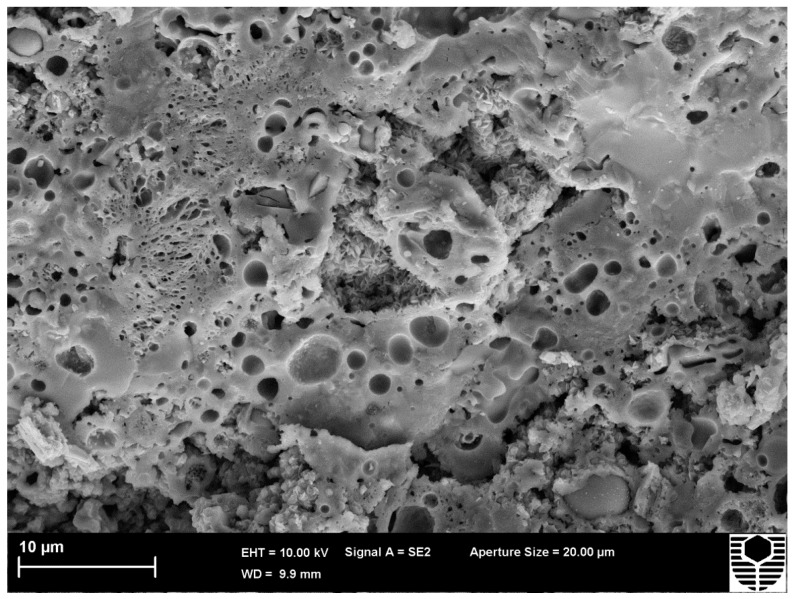
C1.82PP post compression test at 500 °C image.

The increase in compressive strength was from 24.6 MPa at 25 °C to 39.3 MPa at 500 °C. This latter temperature is below that for onset of viscous flow as seen in the dilatometer. However, the applied stress of the test may be sufficient to reduce the activation energy for the viscous flow process. The tenfold increase in strain at maximum load at 500 °C indicates that viscous flow is occurring. The viscous flow is consolidating the microstructure with resulting increases in compressive strength. A comparison of SEM images from as cured (Figure 5) and hot tested (Figure 6) specimens confirms the occurrence of viscous flow during the compression test.

**Figure 7:**
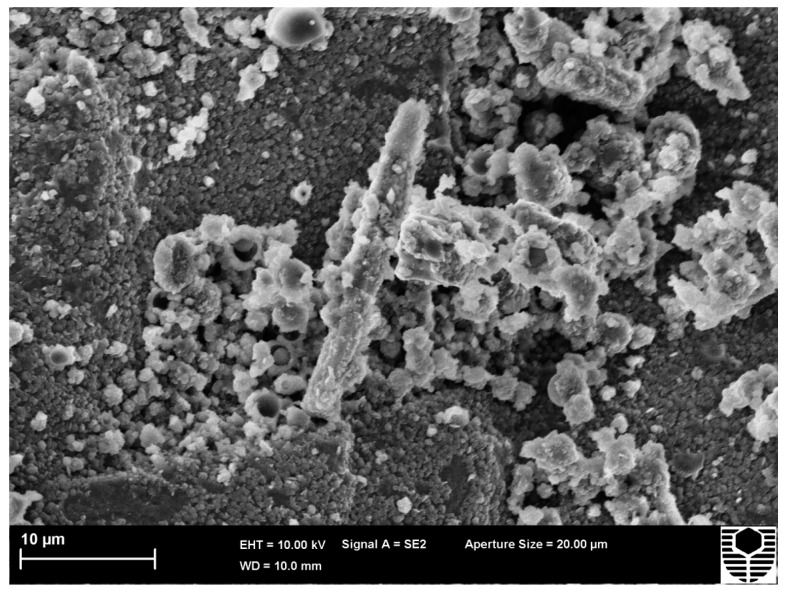
HTC 6A as cured (AC) fracture surface image.

**Figure 8:**
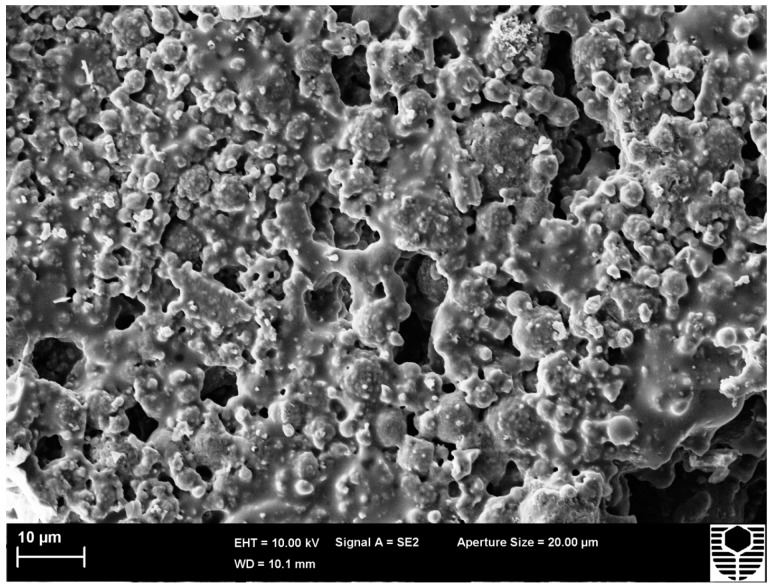
HTC 6A post compression test at 700 °C image.

Testing of HTC 6A at 700 °C (Figure 4) showed an increase in compressive strength from 16.6 MPa obtained at ambient temperature to 35.3 MPa. The change in Young’s modulus is from 10.23 to 2.57 GPa. SEM images, as cured (Figure 7) and tested at 700 °C (Figure 8), again confirm that viscous flow has occurred. The presence of the alumina enables the specimen to support the applied load whilst allowing additional viscous flow to occur after the point of maximum stress has been passed.

### 3.3. Fire Testing

The failure requirements of the AS1530.4-2014 fire test can be summarised as follows:

1. Failure in relation to thermal insulation is determined when measurement of temperature is made by thermocouples on the unexposed face. The specimen is deemed to have failed when:
  (a) Failure criteria 1 (FC1): The average temperature of the unexposed face of the test specimen exceeds the initial temperature by more than 140 K; or
  (b) Failure criteria 2 (FC2): The temperature at any location on the unexposed face of the test specimen exceeds the initial temperature by more than 180 K.
2. Structural adequacy is defined as when a sample either collapses or when the deflection under a given load exceeds that specified in AS1530.4.
3. Integrity failure can show three failure criteria:
  - Continuous flaming on the cold side surface;
  - Through gaps into the furnace, as determined by standard gauges, exceeding the sizes specified in AS1530.4;
  - Ignition during the cotton wool pad test.

**Figure 9:**
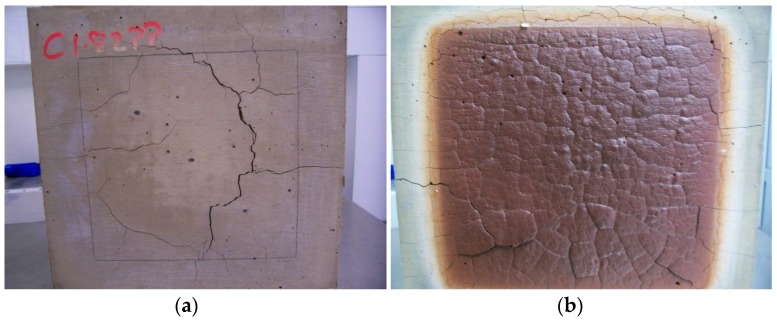
C1.82PP fire test image: (**a**) C1.82PP cold side after fire test; (**b**) C1.82PP hot side after fire test. Some evidence of crack healing is evident. Sample size is 300 mm × 300 mm with region exposed to heat being 200 mm × 200 mm.

**Figure 10:**
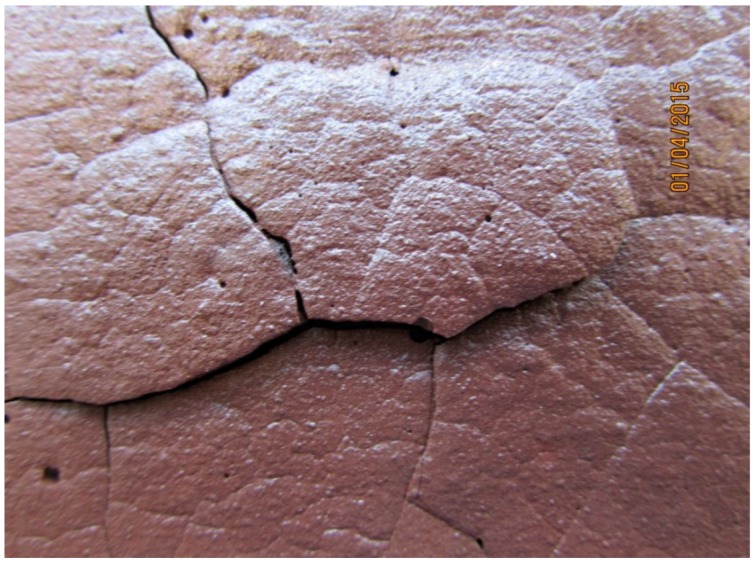
C1.82PP hot side close up showing lifting section attributed to differential thermal expansion/delamination. Healing of other cracks is also evident. Note that this region is clearly visible in Figure 9b and has been enlarged 1.5×.

The paste specimen C1.82PP showed extensive hot side cracking which appeared to have undergone crack healing in some areas on cooling (Figure 9b).This may be attributed to the ability of these unfilled systems to exhibit extensive viscous flow and sintering as seen in Figure 2. The cold side (Figure 9a) formed wide cracks (2 mm) which remained after cooling but did not penetrate the specimen thickness and therefore did not compromise the integrity of the specimen. Figure 10 shows C1.82PP hot side post fire testing where delamination of areas is evident and attributed to differential thermal expansion events in the bulk of the test specimen.

**Figure 11:**
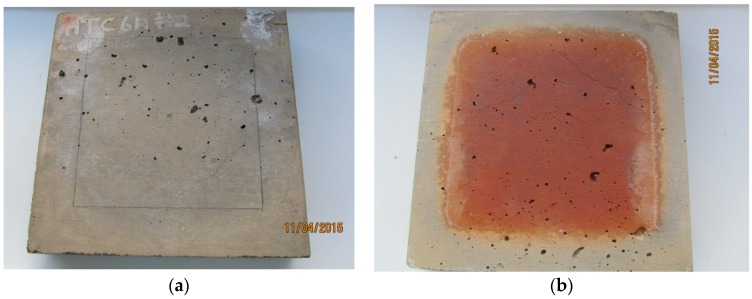
HTC 6A fire test images: (**a**) HTC 6A cold side after fire test; (**b**) HTC 6A hot side after fire test showing few minor cracks.

**Figure 12:**
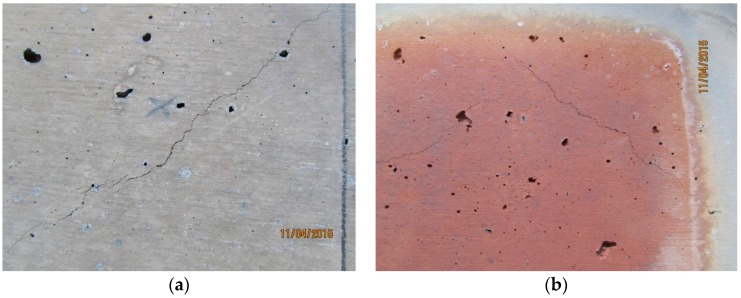
HTC 6A cold side: (**a**) after fire test and hot side; (**b**) after fire test showing fine cracks.

In the case of the specimens with increased loadings of graded alumina, HTC 6A, many large air cavities were evident prior to fire testing. The cavities are attributed to air entrainment which could not be removed by the vibrating table’s lack of power. These cavities were also evident after fire testing. Very few, fine cold side cracks formed during the fire test (Figure 11a and Figure 12a). The hot side showed similar fine crack formation (Figure 11b and Figure 12b). This may be attributed to the low thermal shrinkage values demonstrated by these filled composites. The higher thermal conductivity values for these systems also led to reduced thermal gradients across the thickness of the test specimens and this is clearly shown by the higher cold side temperatures attained during testing. These lower thermal gradients are expected to contribute to a reduced number of and smaller crack formation. The use of the fibre tri-blend would also have contributed to lower crack initiation and growth rates.

**Figure 13:**
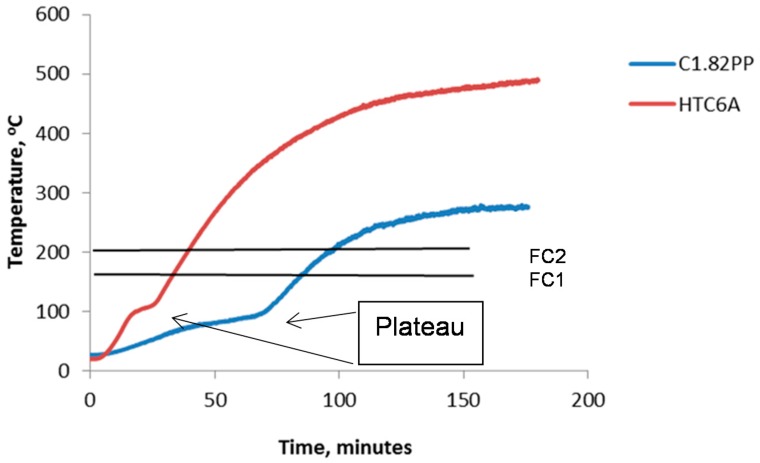
Cold side temperature readings.

The plateau referred to in Table 4 is the “boiling front” which is attributed to the latent heat of evaporation of water. This is illustrated in Figure 13 together with the FC1 and FC2 temperature failure criteria.

The measured weight loss values are lower than the total calculated values as the specimens were allowed to equilibrate at 23 °C and 50% ± 5% relative humidity for at least 28 days prior to fire testing. The decreasing water content (indicated by weight loss in Table 4) as the alumina content increases will lead to shorter plateau durations. Thermal conductivity also increases with decreasing water content but will only play a role up to the end of the dehydration plateau.

The temperature gradient, across the 50 mm thick test slab, for paste is 16 and 12 °C/mm for the aggregate filled test slabs at 120 min into the test. At 180 min the temperature gradients are 16.6 and 12.8 °C/mm, respectively. The lower thermal gradients for the aggregate filled composites would suggest lower thermal stresses are present during the test and together with the lower COTE would produce fewer, smaller cracks. The actual differences between the cold face temperature of paste specimens and aggregate filled composites are in the order of 180 to 200 °C at 120 min into the test and 175 and 205 °C after 180 min. The furnace temperatures for 120 and 180 min are 1049 and 1110 °C, respectively, and were used as reference points for calculating the thermal gradient across the slab.

The temperature increases between 120 and 180 min is in the region of 30 °C for both specimens. After the dehydration plateau has passed the specimens begin to heat up more rapidly depending on the temperature difference between the furnace and the cold side and the thermal conductivity of the slab. The rate of furnace temperature increase is reduced from 104 °C in 30 min (208 °C/h) between the 30 and 60 min test period to 61 °C in 60 min for the 120 to 180 min test period. The thermal conductivity of the systems will also be changing as the temperature increases and the plateau gradient is an indication of the specimen thermal conductivity (higher plateau gradient is related to higher thermal conductivity). The rate of furnace temperature increase appears to have more influence on cold face temperature than thermal conductivity as shown by the narrow temperature increase range of around 30 °C (between 120 and 180 min), across both samples (which will have differing thermal conductivities).

Increasing the volume of alumina in the composites has a marked effect on their response to the fire test. The rapid increase in cold side temperature following the early commencement of the dehydration plateau gives relatively short fire ratings (FC1) compared to paste samples. The addition of alumina increases the thermal conductivity of the composite (see Table 4) and the water content reduces as the alumina content increases (due to a lower paste content). In a separate experiment drying of the composite at 100 and 250 °C also reduced the thermal conductivity which supported the effect of reduced water content. This reduction in water content means that less energy is required to supply the latent heat of evaporation of the water corresponding to a shorter duration of the dehydration plateau.

Whilst the filled composite gave a reduced fire rating, their integrity, as shown by the formation of only small cracks, even after a 3 h fire exposure time, is maintained. This is despite this system reaching a cold side temperature of 476 °C. Examination of the hot side of this specimen also showed minimal cracking. The brown colouration after firing was lighter than the paste due to the lower percentage of binder, and hence less oxidisable iron, in the composites.

## 4. Summary

Preloading of samples during high temperature exposure initiated viscous flow at lower temperatures than seen in unloaded tests, such as the dilatometer. Careful consideration in designing for the use of geopolymers at elevated temperatures is required so that viscous flow is not initiated during service.

The use of thermally stable filler/aggregate (α-alumina in this case) has led to viscous flow occurring at higher temperatures than in paste specimens.

The use of alumina filler in conjunction with a fibre tri-blend has enabled the production of composites showing good integrity after fire testing for 3 h. This has been at the expense of the fire rating defined in AS1530.4 due to the increased thermal conductivity imparted by the added alumina. However, observed cracking on hot and cold faces was minimal.

Increasing additional water content may extend the fire rating, but care must be taken that as cured physical properties are not overly reduced.

The high thermal conductivity shown by these composites may be beneficial when rapid heat dissipation from components is required. It may also be beneficial in the response to thermal shock loading where reductions in thermal gradients are advantageous.

The selection of alternative fillers to alumina, added as a total replacement or as part of a blend with alumina, may be beneficial in increasing the fire rating without impacting on the integrity of the composite.

## Figures and Tables

**Table 1:** Differences in bulk and amorphous Si:Al for Collie fly ash.

Total Oxide (wt %)	Collie Fly Ash
SiO_2_	51.38
Al_2_O_3_	26.90
Fe_2_O_3_	13.20
CaO	1.74
Bulk Si:Al molar	1.62
Amorphous content, %	54.00
Amorphous Aluminosilicates, %	36.29
Amorphous Si:Al molar	1.15

**Table 2:** Formulations of the two mixtures (parts by weight) with total water content and alumina content at the bottom of the table.

Components	C1.82PP Paste	HTC 6A Composite
Collie Fly Ash	100.0	100.0
Alkaline Activating Solution	39.5	39.5
Polypropylene Fibre	0.15	0.15
Basalt Fibre	-	1.0
Wollastonite	-	10.0
Alumina aggregate 3–5 mm	-	175.0
Alumina aggregate 1–2 mm	-	105.0
Alumina aggregate 0.2–0.5 mm	-	70.0
Additional water	-	7.5
**Total Parts by Weight**	139.65	508.15
Water content, wt %	20	7.2
Alumina content, vol%	0	51.0

**Table 3:** Summary of *in situ* test results. Values in parentheses correspond to the least significant figure in the estimated standard deviation to the left.

As Cured Properties	C1.82PP	HTC 6A
Density, g·cm^−3^	1.98 (1)	2.74 (2)
Compressive strength, MPa	24.5 (12)	16.6 (19)
Young’s Modulus, GPa	9.8 (5)	10.23 (50)
Tested at	500 °C	700 °C
Compressive strength, MPa	38.7 (20)	35.3 (18)
Young’s Modulus, GPa	2.1 (1)	2.6 (1)
Density, g·cm^−3^ after 1000 °C	1.89 (1)	2.73 (1)
Weight loss,% after 1000 °C	19.4 (1)	2.9 (1)
Volume shrinkage,% after 1000 °C	11.8 (11)	1.3 (1)

**Table 4:** Summary of fire test results.

Results	C1.82PP	HTC 6A
vol % alumina	0	51
Calculated wt % water	20	7.2
Weight loss, %	12.0	3.2
Start of plateau, min	37.4	15.9
Plateau duration, min	32.5	7.5
Plateau gradient, °C·min^−1^	0.65	1.56
Cold side temperature at 120 min, °C	247	444
Cold side temperature at 180 min, °C	279	476
FC 1, min	87	32.3
